# From AVATAR Mice to Patients: RC48-ADC Exerted Promising Efficacy in Advanced Gastric Cancer With HER2 Expression

**DOI:** 10.3389/fphar.2021.757994

**Published:** 2022-01-05

**Authors:** Zuhua Chen, Jiajia Yuan, Yingying Xu, Cheng Zhang, Zhongwu Li, Jifang Gong, Yanyan Li, Lin Shen, Jing Gao

**Affiliations:** ^1^ Key Laboratory of Carcinogenesis and Translational Research (Ministry of Education/Beijing), Department of Gastrointestinal Oncology, Peking University Cancer Hospital and Institute, Beijing, China; ^2^ Department of Oncology, Tongji Hospital, Tongji Medical College, Huazhong University of Science and Technology, Wuhan, China; ^3^ Key Laboratory of Carcinogenesis and Translational Research (Ministry of Education/Beijing), Department of Pathology, Peking University Cancer Hospital and Institute, Beijing, China; ^4^ National Cancer Center/National Clinical Research Center for Cancer/Cancer Hospital and Shenzhen Hospital, Chinese Academy of Medical Sciences and Peking Union Medical College, Shenzhen, China

**Keywords:** RC48-ADC, gastric cancer, HER2 expression, PDX model, targeted therapeutic agents

## Abstract

RC48-ADC is a novel humanized antibody specific for human epidermal growth factor receptor 2 (HER2)in conjugation with a microtubule inhibitor *via* a cleavable linker. This study was to evaluate the antitumor activity and mechanism of RC48-ADC in gastric cancer (GC) and explore the population that may benefit from RC48-ADC treatment. Four human GC cell lines and nine patient-derived xenograft (PDX) models were exploited to evaluate the antitumor effect of RC48-ADC or trastuzumab treatment *in vitro* and *in vivo*. The expression and phosphorylation of HER2 were assessed by immunohistochemistry (IHC) staining. Critical molecules of downstream PI3K/AKT and cell cycle and apoptosis signaling pathways were detected and quantified by immunoblotting. Combined with preliminary results of preclinical research, three patients with IHC3+, IHC2+/FISH+, and IHC2+/FISH- of HER2 were enrolled to verify the efficacy of RC48-ADC treatment in advanced GC. *In vitro*, RC48-ADC had superior antiproliferative effects in a dose-dependent manner on GC cells, especially on HER2-positive cells. *In vivo*, RC48-ADC exceeded trastuzumab in GC PDX models with HER2 expression, even in models with moderate to low expression of HER2. Further exploration of mechanism showed that RC48-ADC exerted the antitumor effect by inhibiting phosphorylation of HER2, inducing G2/M phase arrest and cell apoptosis in HER2-expressed PDX models. In clinical practice, RC48-ADC had satisfactory efficacy in HER2-positive and HER2 moderately expressed GC patients and demonstrated promising efficacy in HER2-positive patients who have progressed after anti-HER2 therapy. In conclusion, RC48-ADC exerted promising antitumor activity in HER2-positive as well as score of 2+ in IHC and ISH-negative AGC patients after progression of systematic treatment.

## Introduction

Antibody–drug conjugates (ADCs), a conjugation of a monoclonal antibody, a payload cytotoxic agent, and chemical linkers, have emerged as a promising anticancer strategy for the past decades (Abdollahpour-Alitappeh et al., 2019). ADCs have tumor specificity and antitumor potency not achievable with traditional drugs, through the cellular process of antibody–antigen binding on cancer cell surface, endocytosis into the cell, and releasing of cytotoxin (Hafeez et al., 2020). To date, ADCs including Adcetris^®^, Akalux^®^, Besponsa^®^, Blenrep^®^, Enhertu^®^, Lumoxiti^®^, Mylotarg^®^, Polivy^®^, Trodelvy^®^, and Kadcyla^®^ have been approved for cancer therapy by the US Food and Drug Administration.

Trastuzumab is a monoclonal antibody targeting human epidermal growth factor receptor 2 (HER2) that exerts antitumor activity by mediating antibody-dependent cellular cytotoxicity (ADCC), inhibiting of HER2-mediated signal transduction, and shedding of HER2-extracellular domain (ECD) (Hudis, 2007). Trastuzumab has been approved in the treatment of HER2-positive patients with breast cancer (BC) (Slamon et al., 2001; Piccart-Gebhart et al., 2005) and advanced gastric cancer (AGC) (Bang et al., 2010). T-DM1 (Kadcyla^®^), an ADC comprising trastuzumab and the tubulin inhibitor emtansine, which achieves significantly longer median overall survival (OS) and progression-free survival (PFS) in EMILIA (Verma et al., 2012) and TH3RESA (Krop et al., 2017) trials, has been approved for the treatment of HER2-positive metastatic BC patients who previously received trastuzumab. Although T-DM1 was highly effective in HER2-positive gastric cancer (GC) cells and xenografts, the GATSBY study conferred that T-DM1 was not superior to taxane in patients with previously treated HER2-positive AGC (Thuss-Patience et al., 2017). The treatment of patients with HER2-positive AGC resistant to trastuzumab remains an unmet need.

Trastuzumab deruxtecan (DS-8201a) is a novel HER2-ADC composed of trastuzumab and a topoisomerase I inhibitor payload that hasbeen recently approved by the FDA for the treatment of patients with previously treated HER2-positive AGC (Ogitani et al., 2016). The DESTINY-Gastric01 study demonstrated the significant improvements in response and OS of DS-8201a among HER2-positive AGC (Shitara et al., 2020). The objective response rate (ORR) of DS-8201a in patients with immunohistochemistry (IHC)3+ (58%, 53/91) was higher than that in patients with IHC2+ and ISH+ (29%, 8/28), which sparked additional interest of ADCs in therapeutic development of AGC. RC48-ADC is a humanized anti-HER2 monoclonal antibody (hertuzumab) conjugated to microtubule inhibitor monomethyl auristatin E (MMAE) *via* a cleavable linker. A phase 2 study reported that RC48-ADC was well tolerated and demonstrated an ORR of 51.2% in patients with previously treated HER2-positive locally advanced or metastatic urothelial carcinoma (Sheng et al., 2021). This study explored the antitumor effect and mechanism of the RC48-ADC in GC cells and AVATAR models and evaluated its efficacy on three AGC patients with different statuses of HER2.

## Materials and Methods

### Patients and Tumor Samples

This study included three patients with AGC who received systematic treatment from 2017 to 2021 at Peking University Cancer Hospital, Beijing, China. Histopathology confirmation and HER2 detection were determined by two pathologists. This study was approved by the institutional review board at Peking University Cancer Hospital. The clinical response of treatment was evaluated by computed tomography (CT) and was categorized as a complete response (CR), partial response (PR), stable disease (SD), or progressive disease (PD), according to the RECIST 1.1 criteria.

This study was approved by the Medical Ethics Committee of Peking University Cancer Hospital. All animal studies complied with the ARRIVE guidelines and were conducted in accordance with the National Institutes of Health Guide for the Care and Use of Laboratory Animals (NIH Publications No. 8023, revised 1978). Experiments involving human were in accordance with the ethical standards of committees (institutional and national) and with The Code of Ethics of the World Medical Association (Declaration of Helsinki). All patients completed written informed consent prior to study entry.

### Reagents and Antibodies

RC48-ADC was provided by RemeGenCo, Ltd., and dissolved in normal saline. Trastuzumab was purchased from Shanghai Roche Pharmaceutical Ltd. Antibodies specific for HER2, pHER2, AKT, pAKT, S6, pS6, ERK, pERK, pCDK1, CDK2, cyclin E1, p53, Bcl-2, and Bax were purchased from Cell Signaling Technology (Boston, MA, USA). The antibody specific for *β*-actin was purchased from Sigma-Aldrich (St. Louis, MO, USA).

### Cell Lines and Cell Culture

Two HER2-positive GC cell lines (NCI-N87 and SNU-216) and two HER2-negative GC cell lines (NUGC-4 and HGC-27) were used in this study. NCI-N87 was kindly provided by professor You-yong Lv (Peking University Cancer Hospital and Institute, China); SNU-216 and NUGC-4 cell lines were purchased from Cobioer Biological Technology (Nanjing, China). HGC-27 was purchased from the cell bank of Peking Union Medical College (Beijing, China). GC cells were cultured in RPMI 1640 (Gibco, MD, USA) supplemented with 10% fetal bovine serum (Gibco) and then incubated in a humidified incubator (37°C) with 5% CO_2_. All cell lines were confirmed by short-tandem repeat (STR) analysis.

### Cell Viability Assay

A total of 5,000 cells per well were plated onto 96-well plates and incubated with complete medium overnight. Cells were exposed to RC48-ADC (0–10,000 nM) and trastuzumab (0–10,000 nM) for 72 h. The cell viability was assessed by Cell Counting Kit-8 assay (Dojindo, Kumamoto, Japan). The absorbance at 450 nm was measured by a microplate spectrophotometer. All of the experiments were repeated three times. The IC50 was calculated using GraphPad Prism 7.0.

### The Antitumor Activity of RC48-ADC in AVATAR Mice

The establishment and molecular characteristics of AVATAR models for AGC patients were previously reported (Zhu et al., 2015; Chen et al., 2018). Tumor tissues of nine PDX models were subcutaneously inoculated into the flank of 6-week-old non-obese diabetic/severe combined immunodeficiency (NOD/SCID) mice. When the tumor volume reached 750 mm^3^, we separated the tumors, sliced into small fragments, and then reinoculated into other NOD/SCID mice. Mice with tumors of 150–250 mm^3^ were randomized to RC48-ADC group (5 mg/kg), trastuzumab group (5 mg/kg), and vehicle group (physiological saline). All animals were administrated *via* weekly vein injection for 3 weeks. The length and width of tumor tissues and body weights of mice were measured twice a week, and the tumor volume was calculated as (Length × Width^2^)/2. Mice were sacrificed after the administration cycle or when the tumor volume reached 2,000 mm^3^. Tumor growth inhibition (TGI) was determined as [1–ΔT/ΔC] × 100% (ΔT and ΔC presented changes in tumor volume of the treatment group and vehicle group over the course of the treatment).

### Western Blotting Analysis

Total protein was extracted from tumor tissues and the concentration was measured *via* BCA Protein Assay Kit (Beyotime, Shanghai, China). Here, 50 μg protein samples were separated by 10% sodium dodecyl sulfate polyacrylamide gel electrophoresis (SDS-PAGE)and transferred onto nitrocellulose membranes (GE Healthcare, Piscataway, NJ). After incubation with corresponding primary antibodies diluted in 5% bovine serum albumin (BSA)overnight at 4°C and incubation with secondary antibodies for 1 h at room temperature, protein samples were visualized using ECL-plus Western Blotting Detection Reagents (GE Healthcare Life Sciences, Chalfont, UK). Protein bands were quantified and normalized with ImageJ software.

### Immunohistochemistry Staining

Tumor tissues were isolated from euthanized mice, and then formalin-fixed paraffin-embedded (FFPE) tissue blocks were prepared. IHC staining for HER2 was performed according to the manufacturer’s instructions and interpreted by two independent pathologists. IHC scores for HER2 were interpreted as follows: 0, no staining; 1+, weak or focal staining; 2+, moderate staining; and 3+, strong staining.

### Statistical Analysis

The differences between/among groups were analyzed using unpaired two-tailed t-tests, one-way ANOVAs, or factorial analysis by GraphPad Prism version 7.0 (GraphPad Software Inc., CA, USA).

## Results

### RC48-ADC Exerted Selective Antitumor Activity in Gastric Cancer Cells and Patient-Derived Xenograft Models

**FIGURE 1:**
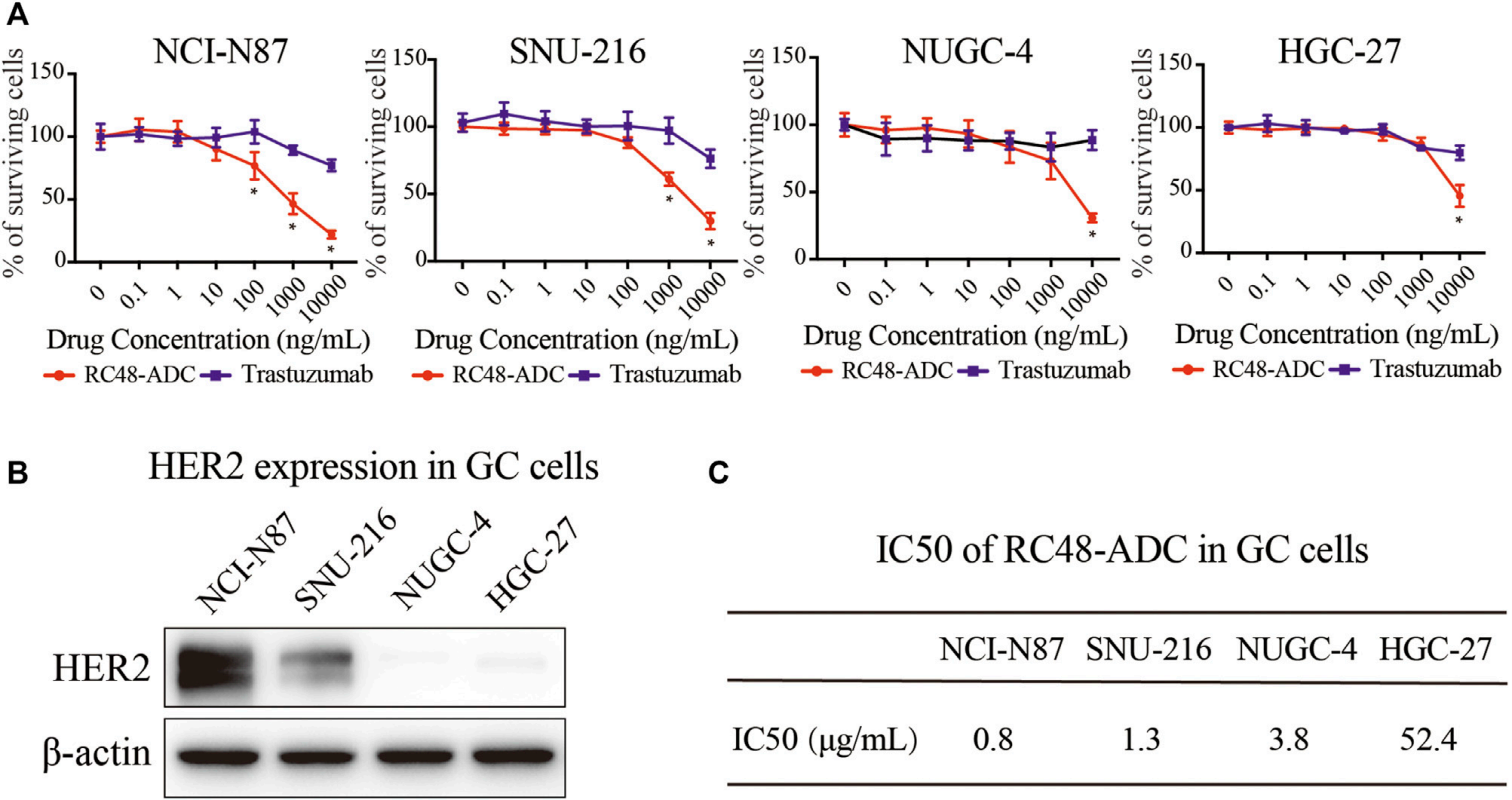
RC48-ADC had superior antiproliferative effects than trastuzumab on 4 gastric cancer (GC) cell lines. **(A)** Cell viability of NCI-N87, SNU-216, NUGC-4, and HGC-27 was detected by CCK-8 assays after RC48-ADC (0–10,000 nM) treatment for 72 h. Data are presented as means ± SD of three independent experiments. **(B)** The expression of human epidermal growth factor receptor 2 (HER2) in 4 GC cells quantified by Western blotting. **(C)** The IC50 of RC48-ADC on GC cell lines evaluated by CCK-8 assay.

Cell viability tests wereconducted to evaluate the antitumor activity of RC48-ADC on 4 GC cell lines, followed by protein expression analysis to clarify the profiling of those cells. Compared with trastuzumab, RC48-ADC had superior antiproliferative effects in a dose-dependent manner on 4 cell lines (Figure 1A). NCI-N87 and SNU-216 cells were more sensitive to RC48-ADC treatment, resulting from the superior expression of HER2 (Figures 1B, C).

**FIGURE 2:**
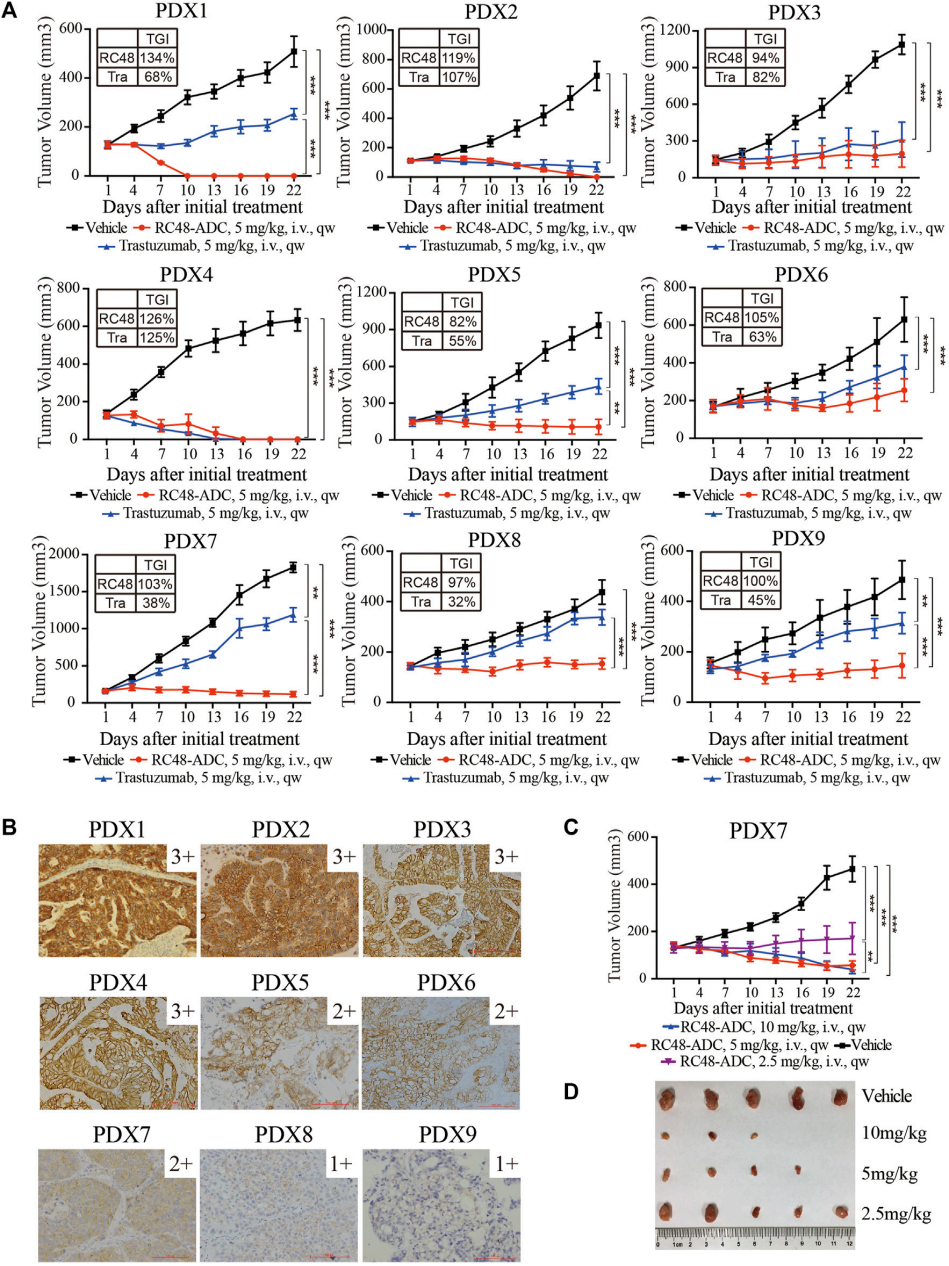
RC48-ADC exerted selective antitumor activity in gastric cancer (GC) patient-derived xenograft (PDX) models with human epidermal growth factor receptor 2 (HER2) expression. **(A)** The antitumor activity of RC48-ADC and trastuzumab treatment in nine PDX models with different expression levels of HER2. Data are presented as means ± SD (*n* = 5 mice per group). TGI, tumor growth inhibition; i.v., intravenous injection. ** p < 0.005, *** p < 0.001 according to repeated-measures ANOVAs. Tra, trastuzumab. **(B)** The expression of HER2 evaluated by immunohistochemistry in PDX models. Scores were interpreted as 3+, 2+, 1+, and 0 (×200 magnification; scale bar represents 100 µm). **(C)** The antitumor activity of RC48-ADC under different concentrations in a PDX model with moderate expression of HER2. ** p < 0.005, *** p < 0.001 according to repeated-measures ANOVAs. **(D)** Tumor size of xenografts in the four groups.

Nine GC PDX models were exploited to evaluate the TGI of RC48-ADC and trastuzumab *in vivo*. In general, RC48-ADC showed excellent antitumor activity in PDX models with different expression levels of HER2 (TGI: 82%–134%, p < 0.05; Figure 2A, Table 1). In PDX models with high expression (IHC3+) and amplification (FISH+) of HER2 (Figure 2B **,** Table 1), the antitumor activity of RC48-ADC was significantly superior to trastuzumab in PDX1 (TGI: 134% vs. 68%, p < 0.001) and equivalent to trastuzumab in three other models (TGI: 94%–126% vs. 82%–125%). As to PDX models with moderate and low expression of HER2, RC48-ADC exerted significantly stronger antitumor efficacy than trastuzumab (TGI: 82%–105% vs. 32%–63%, p < 0.001). The dynamic changes in body weight of mice during treatment were presented as Supplementary Figure S1. In addition, one PDX model with moderate expression of HER2 was chosen to further explore the antitumor activity under different drug concentrations of RC48-ADC. The antitumor effect of 5 mg/kg group exceeded that of the 2.5 mg/kg group, whereas it equaled that of the 10 mg/kg group, which suggested that the efficacy of RC48-ADC was dose-dependent (Figures 2C, D).

**TABLE 1:** The molecular characteristics and tumor growth inhibition of RC48-ADC in PDX models.

PDX ID	HER2 IHC	HER2 FISH	TGI (%)	
			Trastuzumab	RC48-ADC
1	3+	+ (cluster)	68	134
2	3+	+ (cluster)	107	119
3	3+	+ (cluster)	82	94
4	3+	+ (cluster)	125	126
5	2+	− (3:2)	55	82
6	2+	− (1:1)	63	105
7	2+	− (3:2)	38	103
8	1+	− (1:1)	32	97
9	1+	− (1:1)	45	100

### RC48-ADC Decreased the Phosphorylation of HER2 and Induced Cell Cycle Arrest in G2/M Phase and Apoptosis in Gastric Cancer Patient-Derived Xenograft Models

**FIGURE 3:**
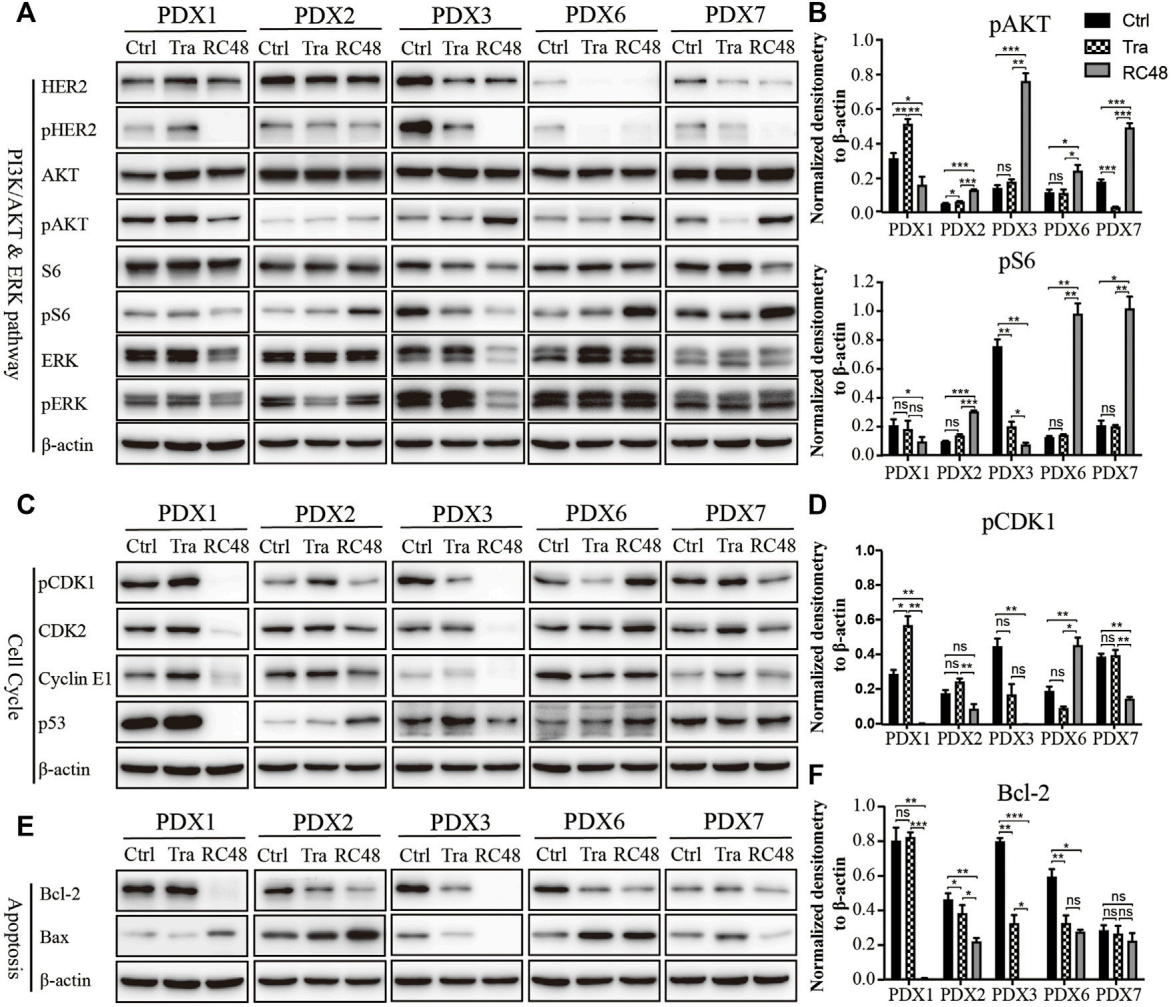
RC48-ADC decreased the phosphorylation of human epidermal growth factor receptor 2 (HER2) and induced cell cycle arrest in G2/M phase and apoptosis in gastric cancer (GC) patient-derived xenograft (PDX) models. **(A, B)** Expression and quantification of critical molecules in the PI3K/AKT/S6 and ERK signaling pathway. **(C, D)** Expression and quantification of critical molecules in the cell cycle pathway. **(E, F)** Expression and quantification of antiapoptotic protein Bcl-2 and Bax. ns, no significance; * p < 0.05; ** p < 0.01; *** p < 0.001 according to repeated-measures ANOVAs. Data are presented as means ± SDs of three independent experiments.

RC48-ADC was a humanized monoclonal antibody specific for HER2 (hertuzumab) conjugated with MMAE. It exerted an antiproliferative effect *via* blocking HER2-driven signaling such as the PI3K/AKT/mTOR and MAPK pathways and inducing cell cycle arrest in G2/M phase through microtubule depolymerization. We detected the protein expression and phosphorylation of HER2 as well as its downstream AKT, S6, and ERK in five PDX models with moderate to high expression of HER2. Both trastuzumab and RC48-ADC could decrease the phosphorylation of HER2, and the inhibitory effect of RC48-ADC was stronger (Figure 3A). In addition, the phosphorylation of downstream AKT and S6 was significantly increased after RC48-ADC treatment in the three PDX models (Figures 3A, B).

As a key regulator in the transition from G2 phase to M phase, CDK1 was found to be involved in modulating the cell cycle by forming the CDK1/cyclin B1 complex. The critical regulatory step in activating cdc2 during progression into mitosis appears to be dephosphorylation of cdc2 at Thr14 and Tyr15. After 3 weeks of RC48-ADC treatment, the phosphorylated CDK1 (Thr14) was downregulated when compared with the vehicle group. Meanwhile, the expression of CDK2 and CyclinE1 also decreased to a certain extent (Figures 3C, D), which further suggested the G2/M cycle block induced by MMAE.

RC48-ADC could also exert its antitumor effect by inducing apoptosis. After treatment with RC48-ADC, the expression of antiapoptotic protein Bcl-2 decreased, accompanied by the upregulation of proapoptotic protein Bax in PDX models (Figures 3E, F).

### RC48-ADC Demonstrated Promising Efficacy in Advanced Gastric Cancer Patients With HER2 Expression

**FIGURE 4:**
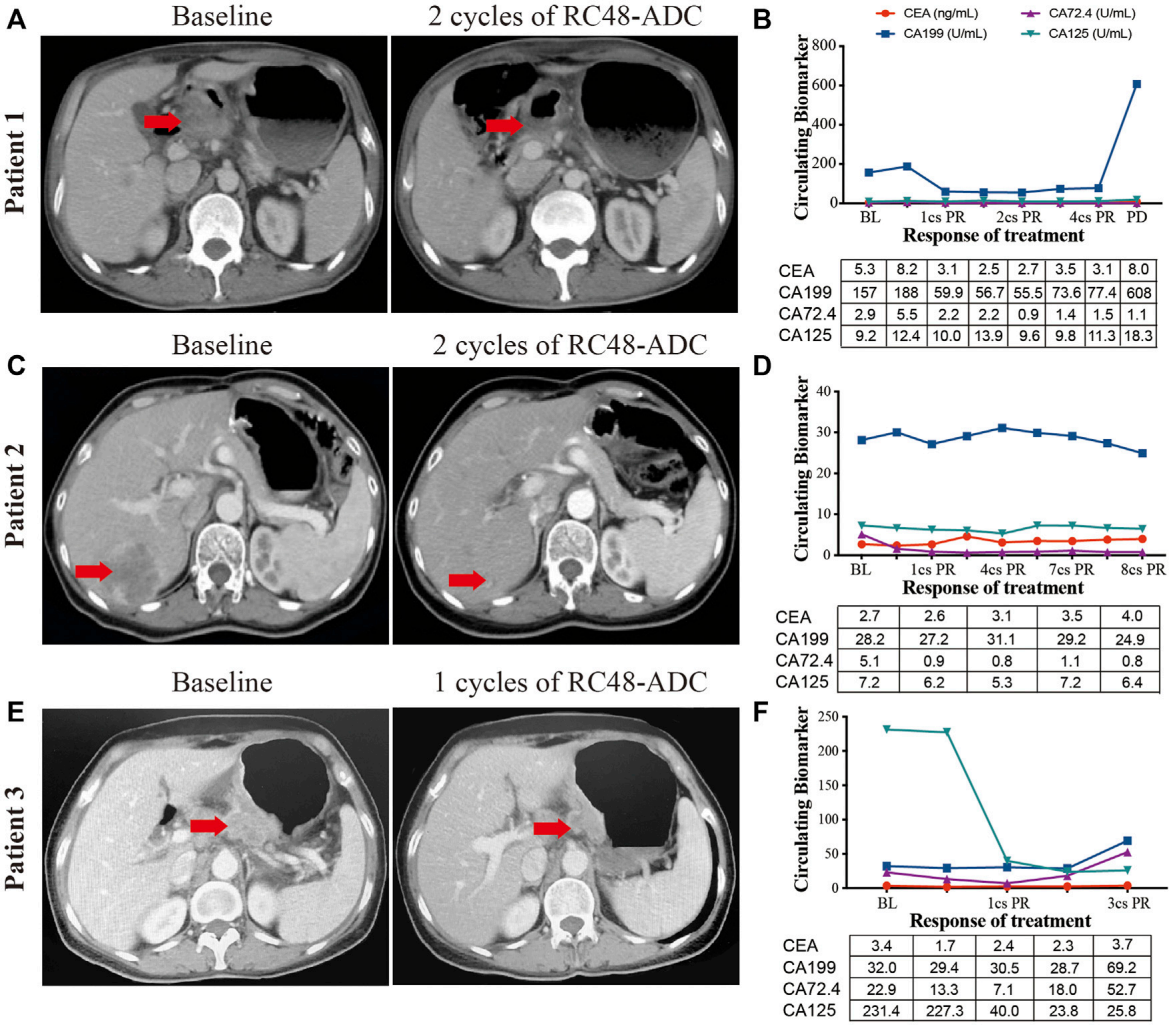
The clinical responses of three human epidermal growth factor receptor 2 (HER2)-expressed advanced gastric cancer (AGC) patients treated with RC48-ADC. **(A**, **C**, **E)** Abdominal CT scans of tumor lesions before and after one or two cycles of RC48-ADC treatment in three patients. **(B**, **D**, **F)** The dynamic change of CEA, CA199, CA72.4, and CA125 during RC48-ADC treatment in three patients. BL, baseline; PR, partial response; SD, stable disease; PD, progressive disease.

Three AGC patients with HER2 overexpression were enrolled to receive RC48-ADC treatment after disease progression with systematic therapies. The characteristics of these patients were shown in Table 2. All of them were administered intravenously with 2.5 mg/kg of RC48-ADC every 2 weeks during a treatment cycle of 6 weeks. Patient 1, a 56-year-old man, was diagnosed as HER2-positive (IHC3+/FISH+) GC with multiple liver metastases. Previously, he was administered five cycles of XELOX regimen in combination with trastuzumab and one cycle of paclitaxel. After two cycles of RC48-ADC treatment, he achieved a clinical response of PR accompanied by decreased CA199 (Figures 4A, B). Treatment-related adverse events (TRAEs) including fatigue (grade 2), diarrhea (grade 1), and neurotoxicity (grade 1) were observed. The disease progressed after five cycles of treatment, and the PFS was 258 days.

**TABLE 2:** The clinical characteristics of the enrolled AGC patients.

Patient	Gender	Age	TNM	Primary site	Metastatic Site	Differentiation	Lauren classification	HER2 IHC	HER2 FISH	Prior therapies
1	Male	56	pT3N2M1	Antrum	Liver	Medium	Mixed	3+	+	1st: XELOX + trastuzumab*5cs
										2nd: Paclitaxel*1cs
										3rd: RC48-ADC*4cs
2	Female	54	ypT4aN0M0→M1	Antrum	Liver	Medium	Intestinal	2+	+	Neoadjuvant: SOX*4cs
										1st: TS*4cs
										2nd: lapatinib + capecitabine*4cs
										3rd: apatinib + trastuzumab*7cs
										4th: RC48-ADC*8cs
3	Female	62	cT + N + M1	Corpus	Peritoneum Ovary	Low	Mixed	2+	−	1st: DP*5cs
										2nd: TS*8 cs, S-1maintained
										3rd: RC48-ADC*4cs

Patient 2 was a 54-year-old woman who was initially diagnosed with HER2-positive (IHC2+/FISH+) GC at stage IIB. She received four cycles of adjuvant chemotherapy with SOX regimen and subsequently underwent gastrectomy. The disease progressed with liver metastasis during the first-line treatment of TS regimen, then she received lapatinib combined with capecitabine for four cycles and apatinib in combination with trastuzumab for seven cycles. After two cycles of treatment with RC48-ADC, the liver lesion obviously reduced in size and resulted in the clinical response of PR (Figures 4C, D). Due to a grade 2 neurotoxicity, the dosage of RC48-ADC was reduced to 2 mg/kg from the fourth treatment cycle. This patient achieved a maintained PR until the last follow-up on June 11, 2021.

Patient 3 was a 62-year-old woman diagnosed with HER2-moderate expressed and FISH-negative GC with peritoneum and ovary metastases. She previously received multiple chemotherapy without anti-HER2 treatment, and the disease progressed during the maintenance therapy of S-1. After one cycle of RC48-ADC treatment, the thickness of the stomach wall obviously decreased in abdominal CT scan accompanied by the decrease of CA125. She achieved PR as the best response and experienced TRAEs including grade 2 fatigue and grade 1 neurotoxicity (Figures 4E, F). She died due to gastrointestinal hemorrhage during the fourth cycle of treatment, and the PFS was 177 days.

The preliminary results show that RC48-ADC has satisfactory efficacy in HER2-positive or HER2-moderate expressed GC patients, and the adverse effects are tolerable. In addition, RC48-ADC has also shown promising antitumor effects in HER2-positive patients who have progressed after receiving anti-HER2 therapy. Among these three patients, the adverse events were fatigue (grade 2), diarrhea (grade 1), and neurotoxicity (grades 1–2).

## Discussion

In the present study, we evaluated the antitumor activity and mechanism of RC48-ADC in GC cells and PDX models and explored its efficacy on three AGC patients with different statuses of HER2. We found that 1) RC48-ADC exceeded trastuzumab in GC PDX models with HER2 expression, even in models with moderate to low expression of HER2; 2) RC48-ADC exerted an antitumor effect by inhibiting the phosphorylation of HER2, inducing G2/M phase arrest and cell apoptosis in HER2-expressed PDX models; 3) RC48-ADC had satisfactory efficacy in HER2-positive and HER2-moderate expressed GC patients and demonstrated promising efficacy in HER2-positive patients who have progressed after anti-HER2 therapy.

Although T-DM1 showed promising efficacy in preclinical research, the GATSBY study conferred that T-DM1 was not superior to taxane in patients with previously treated HER2-positive AGC (Thuss-Patience et al., 2017). Unlike the conjugation of trastuzumab and the tubulin inhibitor emtansine in T-DM1, RC48-ADC is composed of hertuzumab and the microtubule inhibitor MMAE. Compared with trastuzumab, hertuzumab was reported to have a higher affinity to HER2 and capacity of antibody-dependent cell-mediated cytotoxicity (ADCC) *in vitro* (Li et al., 2016). After conjugation with MMAE, the cytotoxicity of hertuzumab was significantly enhanced, whereas the binding specificity for HER2 was not affected (Li et al., 2016). Furthermore, unlike T-DM1 with minimal bystander effect on nearby cells due to poor membrane permeability, RC48-ADC has a bystander effect that can reverse T-DM1 resistance by acting on populations of cells not overexpressing HER2 (Staudacher and Brown, 2017). Preclinical study showed that RC48-ADC exerted much stronger antitumor activity than monotherapy of trastuzumab, hertuzumab, MMAE, and combination treatment of hertuzumab and MMAE in NCI-N87 xenograft models (Li et al., 2016). Based on these published results, we evaluated the efficacy of RC48-ADC on GC cells, AVATAR mice, and patients in the present study.

Consistent with previous studies, we observed the superior antitumor activity of RC48-ADC than trastuzumab in GC cells and PDX models. Recently, the series of DESTINY study revealed that the ORR of DS-8201a was higher among patients with IHC3+ than those with IHC2+ and ISH-positive patients (Shitara et al., 2020; Siena et al., 2021), which suggests higher levels of HER2 expression seem to result in a better response. In the present study, all the four HER2-positive PDX models were confirmed with score of 3+ on IHC analysis. Due to the lack of model with 2+ of IHC and positive result on ISH, we could not evaluate the efficacy of RC48-ADC on those PDX models. Previous research reported that higher HER2 expression was associated with enhanced uptake and intracellular release of conjugated MMAE (Li et al., 2020), which might explain the difference in efficacy among HER2-positive patients with different expression levels.

Another finding of this study was that RC48-ADC exerted a promising antitumor activity in models with moderate to low expression of HER2 and achieved the clinical response of PR in a previously treated patient with 2+ of IHC and negative result on ISH. According to literature, patients with HER2 IHC2+/FISH− account for about 40%–60% of GC (Liu et al., 2016), which is expected to expand the targeted population of RC48-ADC. Actually, in a phase I study of RC48-ADC that we conducted in advanced solid tumors, patients with HER2 IHC2+/FISH- (ORR: 5/14, 35.7%) responded similarly to those with IHC2+/FISH+ (ORR: 2/10, 20%) and IHC3+ (ORR: 3/22, 13.6%) (Xu et al., 2021). In addition, a phase II study of RC48-ADC also reported that eight urothelial carcinoma patients with IHC2+ and FISH-negative experienced PR (ORR: 40%) (Sheng et al., 2021). Combined with these preliminary results in preclinical and early clinical research, we further designed and conducted a single-arm phase II study to explore the efficacy and safety of RC48-ADC for patients with HER2-overexpressed AGC (NCT03556345). In 125 enrolled patients, the ORR of RC48 was 24.8% (31/125). The median PFS and OS were 4.1 months (95% CI: 3.7–4.9 months) and 7.9 months (95% CI: 6.7–9.9 months), respectively. Furthermore, the ORR of RC48-ADC in patients with HER2 IHC2+/FISH- (1/6, 16.7%) is lower than that in HER2-positive patients (20/76, 26.3%) (Peng et al., 2021). In June 10,^,^ 2021, RC48-ADC was approved by the National Medical Products Administration for the treatment of locally advanced or metastatic GC with HER2 overexpression who had received at least second-line treatment of systemic chemotherapy. A randomized controlled phase III trial is ongoing to compare the efficacy and safety of RC48-ADC with those of the current third-line treatment of AGC patients (NCT04714190).

There are some limitations in this study. On one hand, the efficacy comparison of RC48-ADC, T-DM1, and DS-8201 in cell lines and PDXs was not conducted. From the results of phase II studies (NCT03556345 and NCT03329690), the ORR of RC48 (24.8%, 31/125) is relatively lower than that of DS-8201 (51%, 61/119) (Shitara et al., 2020; Peng et al., 2021). Considering the differences in the baseline characteristics of the enrolled patients in the two studies, the difference in efficacy between RC48 and DS-8201a needs to be further explored. On the other hand, due to the lack of model with IHC2+/ISH+ of HER2, we could not compare the efficacy of RC48-ADC in these models with HER2 IHC3+ models.

## Conclusion

RC48-ADC exerted promising antitumor activity in HER2-positive as well as IHC2+ and ISH-negative AGC patients after progression of systematic treatment.

## Data Availability

The original contributions presented in the study are included in the article/Supplementary Material, further inquiries can be directed to the corresponding authors.
