# Supplementary figures and images for: From AVATAR Mice to Patients: RC48-ADC Exerted Promising Efficacy in Advanced Gastric Cancer With HER2 Expression
Source: Front Pharmacol. 2022 Jan 5;12:757994. doi: 10.3389/fphar.2021.757994 (PMC8769204; doi:10.3389/fphar.2021.757994)

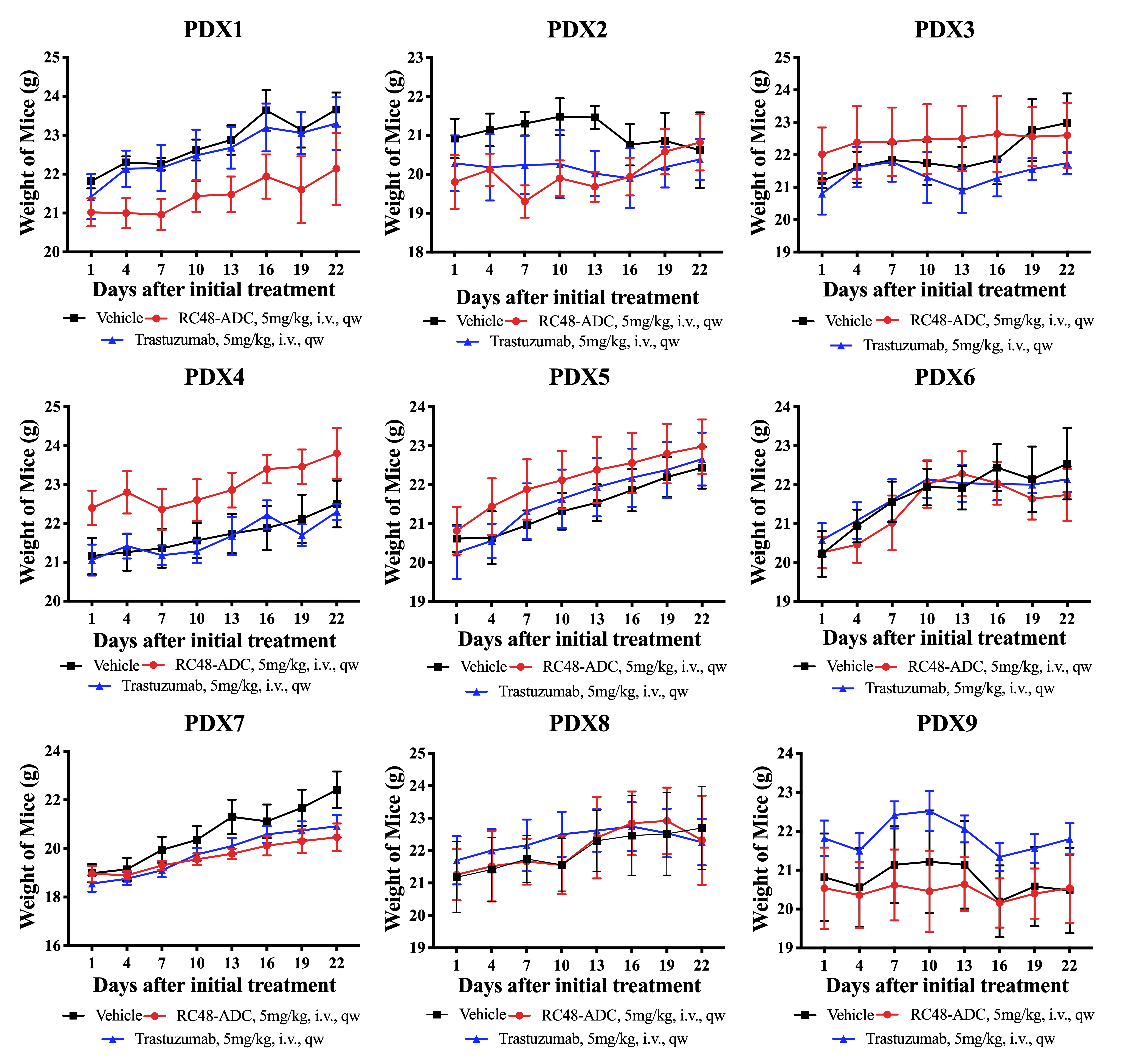

Supplement: Supplementary file 1 [file Image1.TIFF]
